# Evaluation of China-ASEAN trade status and trade potential: An empirical study based on a gravity model

**DOI:** 10.1371/journal.pone.0290897

**Published:** 2023-09-01

**Authors:** Huafeng Zhai

**Affiliations:** Department of Digital Trade, Hangzhou Wanxiang Polytechnic, Hangzhou, Zhejiang, China; University of Economics Ho Chi Minh City, VIET NAM

## Abstract

**Objective:**

The objective of this study was to identify factors influencing the development of China-ASEAN trade- from the total economic volume of both sides, distance, the population size of ASEAN countries, the construction of a free trade area, and the signing of the Belt and Road initiative, resource endowment per capita, the exchange rate between RMB and ASEAN countries, and the land area of ASEAN countries—to develop a conceptual framework for China-ASEAN trade potential.

**Study design:**

This study uses panel data from 2001 to 2021 that is evenly distributed among 10 ASEAN countries to serve as the dataset. Firstly, the unit roots are checked and the cointegration relationships are examined, focusing on the heterogeneity test. Based on the classical trade gravity model, the innovative trade gravity model with key influencing factors is constructed. On the basis of the classical trade gravity model, an innovative trade gravity model of key influencing factors is constructed. The trade potential model is used to calculate the direct trade potential coefficient between China and ASEAN countries, which points out the direction for the sustainability of bilateral trade.

**Results:**

This study finds that among the factors affecting China-ASEAN bilateral trade, the total economic output of both sides, distance, population size of ASEAN countries, the construction of the FTA, and the signing of the Belt and Road Initiative all have a positive impact on bilateral trade. Three influencing factors, namely per capita resource endowment, exchange rate between RMB and ASEAN countries, and the size of ASEAN countries, have a negative impact on bilateral trade, but to a lesser extent. The trade potential between China and Vietnam falls into the category of potential re-modelling, indicating that both sides are currently utilizing their trade potential to the greatest extent possible, that trade growth space is limited, and that new trade opportunities must be discovered. The trade potential index between China and nine ASEAN countries, excluding Vietnam, is in the potential-exploiting category, indicating that the potential has not been fully utilized by both sides and that there is still room for growth in the scale of trade between the two countries.

**Conclusion:**

With the shift of the world’s economic center of gravity in the direction of Asia following COVID-19, China and ASEAN countries should seize the opportunity to strengthen their comprehensive strength and economic aggregates and further develop China’s constructive role in the regional organization. The signing of the Belt and Road Initiative and the construction of a free trade zone has had a positive effect on the development of bilateral trade. Propose that: positive trade factors should continue to be strengthened, trade barriers should be removed, and new dynamics of bilateral trade growth should be enhanced.

## 1. Introduction

Since 1991, China-ASEAN cooperation has been on an upward trajectory, moving from being a comprehensive dialogue partner to a good-neighborly and trusting partner for the 21^st^ century, to a strategic partner for peace and prosperity, and now to a comprehensive strategic partner [1]. For more than 30 years, China has always attached importance to mutually beneficial cooperation with ASEAN. It was the first major country to accede to the Treaty of Amity and Cooperation in Southeast Asia [2]. Data show that in 2022, the two sides released the China-ASEAN Comprehensive Strategic Partnership Action Plan (2022–2025) and the Joint Statement on Strengthening China-ASEAN Joint Sustainable Development, indicating closer political ties, more dynamic economic and trade cooperation, and deeper humanistic exchanges [3]. According to economic forecast data released by the World Bank and several other international institutions, the overall economic situation of ASEAN member countries is positive in 2023, with all ASEAN member countries expected to have positive economic growth [4, 5]. Meanwhile, the Chinese economy is also expected to improve overall in 2023 as China’s epidemic prevention and control measures continue to be optimized, and China and ASEAN are each other’s important investment partners [6].

**Fig 1:**
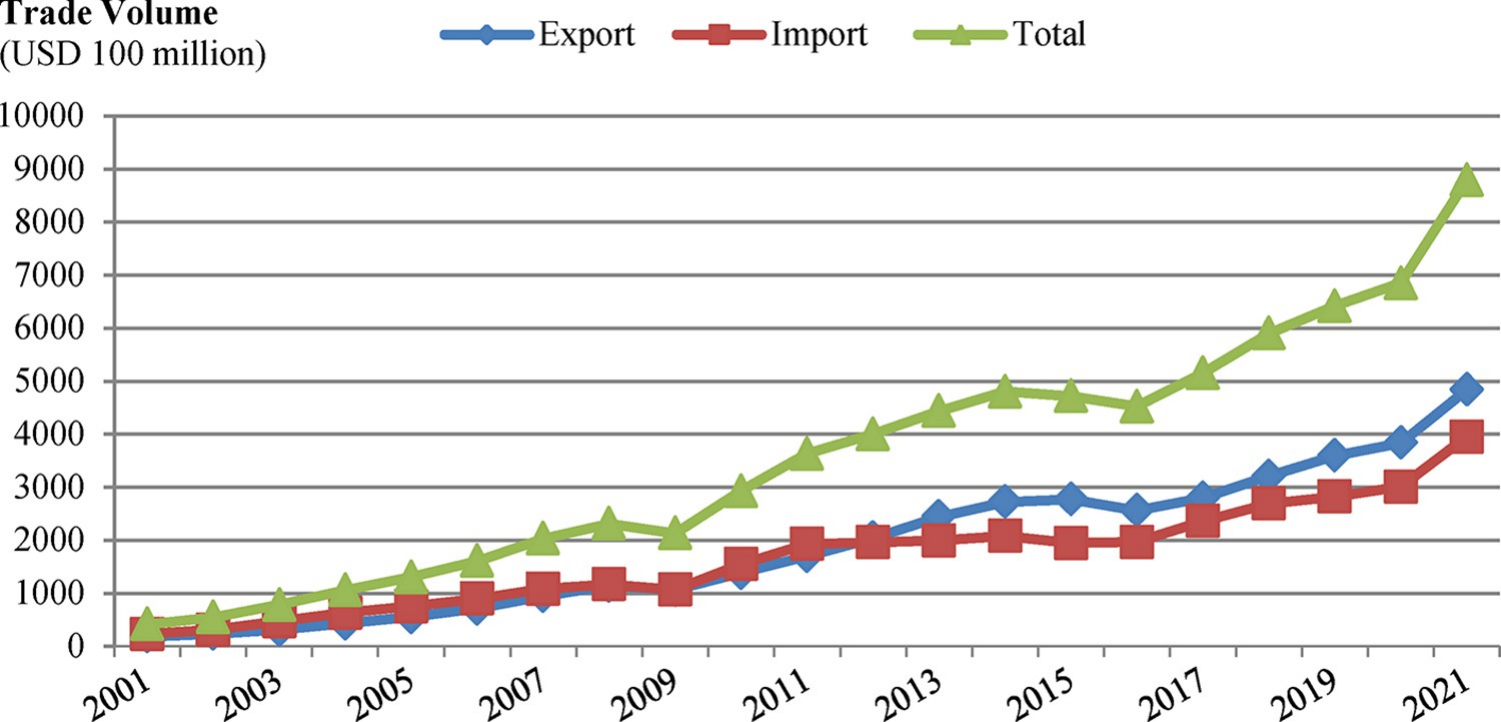
Trade volume (2001–2021). **Source:** UN Comtrade Database (SITC, Rev3) data calculations.

The total value of China’s imports and exports with ASEAN in 2021 will be approximately $878.207 billion, an increase of 28.1% over the previous year. The trade between the two sides is developing rapidly and growing at a strong rate [7]. Looking at the 20-year trend of bilateral trade volume, there were two time periods with a slight decline, namely 2009 and 2015–2016. The decrease in bilateral trade volume in the two years 2015–2016 was mainly due to the following aspects: (1) the overall world economic downturn caused by the general environment; (2) the decline in commodity prices, especially for major trade products between China and ASEAN such as oil, natural gas, rice, and rubber, which led to a decrease in bilateral trade volume; (3) China’s domestic industrial restructuring has led to a reduction in imports of bulk commodities such as minerals; for example, China has reduced its imports of coal from Indonesia and Vietnam [8]. Fig 1 shows the bilateral China-ASEAN trade volume from 2001–2021.

At present, China-ASEAN have become each other’s largest trading partners, yet little is known about what factors may affect the development of China-ASEAN trade, and even less is known about the future trade potential of China-ASEAN. While much research is dedicated to assessing the affect the development of China-ASEAN trade, few investigations explore the strength and differences in the effects of these influences.

This study focuses on (1) using a total of 21 years of bilateral trade import and export data from 2001–2021, it applies the gravity model to assess the influence of various factors; (2) assessing China-ASEAN trade potential using a trade gravity model, which is potentially the most efficient method for trade potential estimation. Such objectives intend to provide support for further deepening China-ASEAN regional cooperation and point the way for both sides to improve their trade structure, enhance trade levels, and formulate trade policies.

With the development of institutional economics, increasing attention has been paid to the impact of formal and informal systems on international trade flows. This study lacks analyses of factors such as the legal system of the trading partner country, the contract implementation guarantee system, and the security of property rights, and will strengthen the exploration of transaction security in future studies.

## 2. Literature review

### 2.1. Trade development between China and ASEAN

Scholars around the world have explored the political and economic spheres more comprehensively, focusing on two main topics. Firstly, exploring the process of economic integration in the Asia-Pacific. The China-ASEAN trade relationship is a practical necessity for the integration of economic cooperation mechanisms within the Asia-Pacific region, as well as a strategic necessity to balance the US-led economic cooperation in East Asia [9]. At present, economic cooperation in the Asia-Pacific region is characterized by "multiple frameworks and competitive cooperation", and China must deal with the different rules under different FTAs, otherwise the "spaghetti bowl effect" will easily occur. The Asia-Pacific region is the core of US economic and strategic interests, and the Trans-Pacific Partnership (TPP) proposed by the US after its high-profile return to the Asia-Pacific region is gradually becoming a strategic cornerstone for the US to dominate the political and economic landscape of the Asia-Pacific region in the future, with ASEAN being seen as one of the most prominent battlegrounds between the two economic powers (the US and China). Strong China-ASEAN trade relations will enhance the sense of community of destiny among countries in the Asia-Pacific region and promote the establishment of the East Asian economic sphere and the building of regional integration in the region. Secondly, predict the economic effects of the rapid development of China-ASEAN trade and welfare changes. The main objective of traditional RTAs is to improve the welfare of members in the region by removing trade barriers to facilitate trade creation and trade transfers. Moreover, scholars have empirically analyzed the economic effects of China-ASEAN trade barrier reduction on member countries using the global trade analysis model (GTAP) and found that China-ASEAN trade will improve member countries’ welfare to varying degrees [10].

An extensive literature search of significant articles and books on FTA and ASEAN was conducted. The economic integration of ASEAN, which has created an integrated market through free trade agreements, is a successful example of economic integration for developing countries, and other developing countries can learn from ASEAN’s experience [11]. ASEAN initiated and led the negotiation of the regional comprehensive economic partnership (RCEP), which is of great significance in ASEAN and East Asia and has ensured ASEAN’s centrality in East Asian economic integration [12]. India has moved towards the regional trade route since 2004, primarily owing to the slow progress of the Doha Round negotiations. The India-ASEAN trade patterns and changing dynamics are assessed and comments on the future of the RCEP [13]. RCEP and the changing world trade landscape are having a positive impact on investment policies and flows in the Asia-Pacific region [14]. The determined negotiation and implementation of a coherent legal framework within ASEAN is important for the ASEAN countries, their neighbors and their trading and investment partners [15].

### 2.2. Empirical studies on trade potential

Zhang and Wang [16] constructed a bilateral export equation for China and used it to calculate an index of China’s trade potential for exports to ASEAN member countries. Bano, Takahashi, and Scrimgeour [17] analyzed the trade potential and main trade patterns between New Zealand and ASEAN. Devadason and Chandran [18] found that the China-ASEAN trade relationship is multi-dimensional. The results of the analysis reflect the main factors influencing the efficiency of China-ASEAN trade. Li and Gu [19] analyzed the scale, structure, and potential of trade between China and the countries along the Belt and Road. It is a rational choice to upgrade the structure of agricultural trade and optimize the environment of the trade to achieve a win-win situation. Shanshan, Zeqi, Jiaqi, and Xiaofeng [20] conducted an empirical analysis of the factors influencing agricultural trade and trade potential between China and ASEAN countries using an extended gravity model.

### 2.3. Empirical studies utilized the trade gravity model

Carrere [21] used a gravity model to assess RTAs. Cheng and Wall [22] used fixed effects to control for heterogeneity. The study shows that integration can have an impact on trade volumes. Burger, Van Oort, and Linders [23] extended the empirical model proposed by Santos Silva and Tenreyro (2006) to propose a log-normal and standard Poisson specification scheme for the trade gravity model. Oguledo and Macphee [24] derived a new gravity model from a linear expenditure system. Gomez-Herrera [25] found that the Heckman sample selection model performed better for the gravity equation. Porojan [26] found that inherent spatial effects have an impact on trade flows. Gravity models show trade activities between countries due to their relative advantages [27].

Such prior empirical research exhibits two key characteristics: Firstly, a large number of scholars have focused on the commodity structure and geographical orientation of trade, the welfare and relief of trade, and the environmental effects of trade. The methods have also been mostly qualitative analysis and indicator accounting, with relatively little econometric analysis. Secondly, insufficient attention has been paid to the commodity structure and trade characteristics of China-ASEAN partner countries, and there is a lack of systematic, product classification-based studies on trade and commodity structure.

While much research is dedicated to assessing the trade status and trade potential, few investigations explore the multifaceted influences on bilateral trade flows, and the extent of the influences. However, there is no comprehensive assessment of the impact of specific key factors, and consequently on China-ASEAN trade status and trade potential. In this paper, I first develop a conceptual framework that describes the pathways from different specific key factors and their characteristics, to the extent of identifying influencing factors, to acquiring the final trade gravity model. I then use this framework to explore the current status of China-ASEAN trade potential and projections for future trade development.

Thus, to provide a comprehensive analysis of the trade and commodity structures of the most populous, largest economic and trade region in the world. This study used the UN Commodity Trade Database to analyze in detail the evolution of the trade and commodity structures of China-ASEAN partner countries, while simultaneously introducing variables such as per capita resource endowment and trade barriers to comprehensively explore the trade patterns and structural characteristics of China-ASEAN partner countries, and employing the gravitational force model to explore the main influencing factors of bilateral trade flows. The aim is to provide a scientific basis for decision-making on trade cooperation in the free trade area.

## 3. Methodology

### 3.1. Dataset and sources

This study uses panel data from 2001 to 2021 that is evenly distributed among 10 ASEAN countries to serve as the dataset. Data was obtained with the best possible uniformity. The explanatory variables are GDP, population size, geographical distance expressed as a product of the price of Brent spot crude oil, resource endowments per capita, the RMB exchange rate with ASEAN countries, ASEAN countries’ land area, FTA, and signing of the Belt and Road cooperation initiative. Data from the World Bank database, WDI, UN Comtrade, China-ASEAN Free Trade Area Network, and China Belt and Road Network, where import dependency is the ratio of annual imports of the importing country to GDP over the same period. Table 1 shows sources of data and descriptions of variables used in the model.

**Table 1:** Sources of data and descriptions of variables used in the model.

Variable	Meaning	Predictive Symbols	Data Source
*lnGDP*_*t*_	China’s GDP in period t	+	WDI
*lnGDP*_*jt*_	GDP of ASEAN member countries in period j	+	WDI
*lnDist*_*ij*_	Distance between China and ASEAN Capital	-	CEPII GeoDist BP World Energy Statistics Yearbook 2022
*lnPOP*_*jt*_	Time j, the population size of ASEAN countries	+	WDI
*lnDG*_*ijt*_	Differences in resource endowments per capita between countries i and j in period t.	-	UN Comtrade
*lnEC*_*ijt*_	Exchange rates between China and ASEAN countries	-	UN Comtrade
*lnLand*_*j*_	j Country land area, as a proxy variable for national resource endowment.	-	CEPII
*BR*_*ij*_	Signing the Belt and Road Initiative. It’s taking 1, not 0	+	"Belt and Road" Web
*FT*_*ij*_	Whether to establish a free trade area. Yes takes 1, No takes 0	+	China-ASEAN FTA WEB

### 3.2. Ethical considerations

Given the data gathering process employed by panel data collection, there were no human participants involved; hence, no consent was acquired.

### 3.3. Conceptual framework

**Fig 2:**
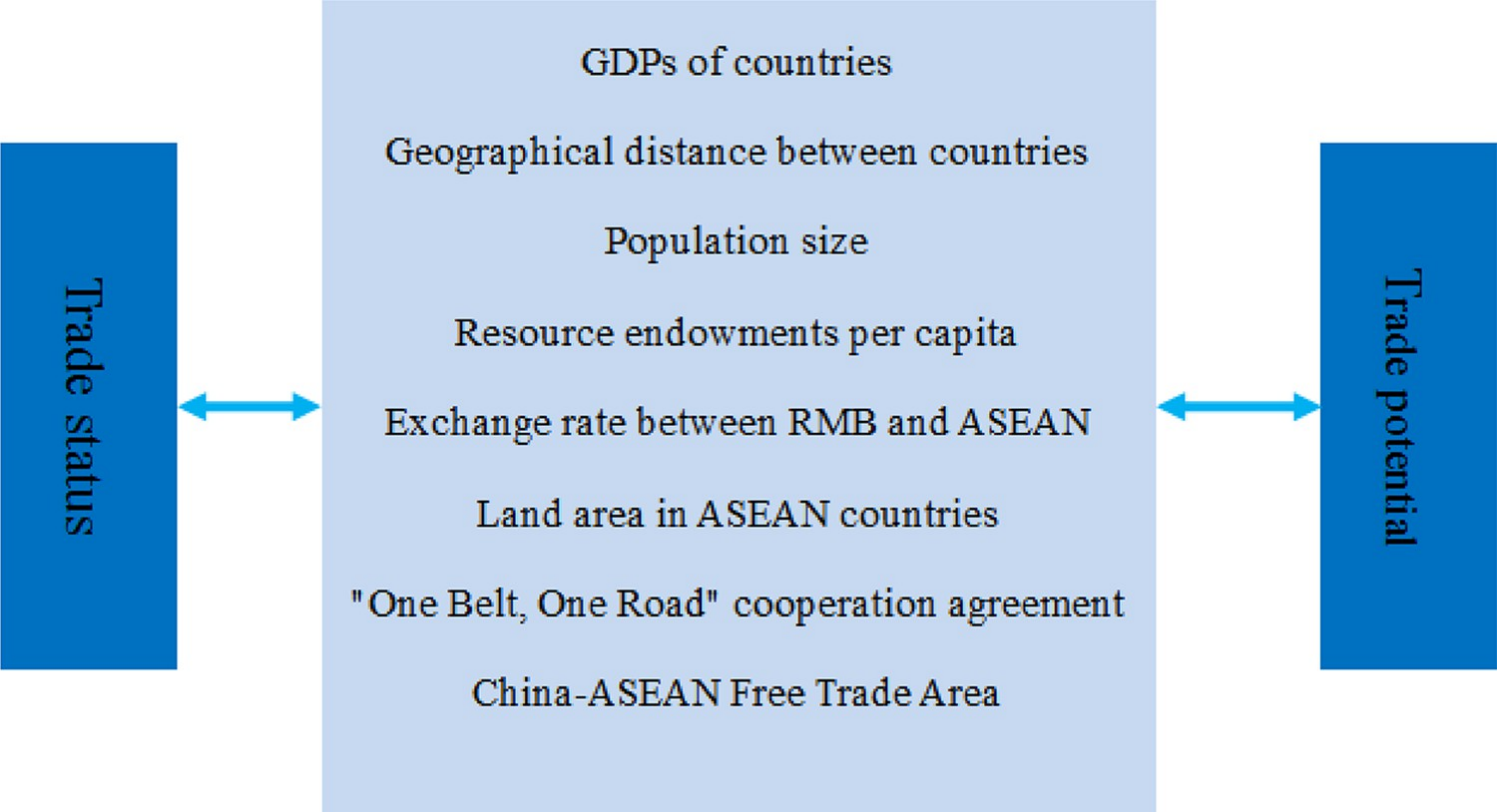
Conceptual framework for trade gravity and trade potential model.

An extensive literature search was conducted to retrieve studies examining the role of trade gravity and trade potential model. This search consisted of a mix of structured searches of electronic databases. Based on my findings a composite conceptual framework was proposed, in which bilateral trade flow is conceptualized as a function of the GDPs of countries, geographical distance between countries, and population size (Fig 2). The framework emphasizes the interaction of factors within and across these levels of influence. All of these factors may directly or indirectly influence bilateral trade flow. In addition to the more traditional influencing factors, the following specific key factors were identified for my study: resource endowments per capita, exchange rate between RMB and ASEAN countries’ currencies, land area in ASEAN countries, "One Belt, One Road" cooperation agreement and China-ASEAN Free Trade Area. Furthermore, my findings indicated that the influence of these factors on bilateral trade flow may differ. This multilevel, interactive framework is useful for understanding and explaining the factors influencing China-ASEAN trade status and trade potential.

### 3.4. Data analytical models

To analyze the data, this study utilized the trade gravity model and the trade potential model, as well as descriptive statistical analysis models.

#### 3.4.1. Descriptive statistical analysis models

Descriptive statistical analysis models such as percentages, mean values, t-tests and F-tests of variables used in the trade gravity and the trade potential model.

#### 3.4.2. Trade gravity model

The gravity model is a widely recognized approach to analyzing global trade trends [28]. In 1962 Jane Tinbergen [28] suggested that the scale of bilateral trade between any two countries is proportional to their respective GDP and geographical distance. In 1998, Deardorff [29] argued that the gravity model could be derived from traditional trade influencing factors. In 2002, Eaton and Cottim [30] derived the trade gravity equation from a Ricardian type of model, and in 2008, Helpman [31] derived a similar trade gravity equation from a theoretical model of international trade. The classical trade gravity model is:

$$T_{ij}=A(GDP_{i}\times GDP_{j})/Dist_{ij}$$ (1)

In Eq (1): T_ij_ is the bilateral trade flow. GDP_i_ and GDP_j_ are the GDPs of countries i and j. A is a constant; Dist_ij_ is the geographical distance between i and j. To ensure the stability of the relevant variables and reduce heteroscedasticity, the log-linear equation is obtained by taking the natural logarithm of all variables in Eq (1) and then regressing them by the least squares method. The estimated equation at this point is:

$$lnT_{ijt}=\beta_{0}+\beta_{1}lnGDP_{it}+\beta_{2}lnGDP_{jt}-\beta_{3}lnDist+\varepsilon_{i}{}_{j}$$ (2)

In Eq (2), T is the bilateral trade flow. i, j represents the trading country, t is the year, β_0_ is the constant and ε_ij_ is the disturbance term. The negative sign in front of β3 can be seen as a hindrance to the distance variable being the bilateral trade volume. It is possible to variant Eq (3) as follows:

$$lnT_{ijt}=\beta_{0}+\beta_{1}lnGDP_{it}+\beta_{2}lnGDP_{jt}+\beta_{3}lnDist+\varepsilon_{i}{}_{j}$$ (3)

With the advance of the globalized economy and the deepening of regional economic integration, bilateral trade flows between countries are no longer influenced solely by the size of their economies and spatial distance. Therefore, to study the factors influencing China-ASEAN trade, this paper extends the traditional gravity model accordingly, and introduces the following variables into the basic form of the gravity model according to the characteristics of China-ASEAN trade and the actual situation of import and export, after fully considering the supply and demand of the country and the trading countries.

Due to the impact of national population size on national productive capacity and international market dependence, the population size variable (POP) is added to the gravity model to obtain Eq (4).


$$lnT_{ijt}=\beta_{0}+\beta_{1}lnGDP_{t}+\beta_{2}lnGDP_{jt}+\beta_{3}lnDist+\beta_{4}\ln POP_{jt}+\varepsilon_{ijt}$$ (4)

In many empirical analyses, scholars usually use the absolute value of the difference in GDP per capita to test the impact of Linde’s Theorem on bilateral trade. The smaller the difference, the more similar the standard of living of the people in the two countries and the more similar their demand preferences are, making it easier to increase trade flows between the two countries. The difference in resource endowments per capita is shown in Eq (5).


$$DG_{ijt}=|\left(GDP_{it}/POP_{it})-(GDP_{jt}/POP_{jt}\right)|$$ (5)

The difference in resource endowment per capita (DG) is added to the gravity model, as in Eq (6).


$$lnT_{ijt}=\beta_{0}+\beta_{1}lnGDP_{t}+\beta_{2}lnGDP_{jt}+\beta_{3}\ln Distij+\beta_{4}\ln POP_{jt}+\beta_{5}{\mathrm{lnDG}}_{\mathrm{ijt}}+\varepsilon_{ijt}$$ (6)

The development of trade between China and ASEAN countries is also affected by exchange rate fluctuations on both sides, therefore, a dummy factor EC for the exchange rate between RMB and ASEAN countries’ currencies is introduced in the gravity model, as shown in Eq (7).


$$\begin{array}{l} lnT_{ijt}=\beta_{0}+\beta_{1}lnGDP_{t}+\beta_{2}lnGDP_{jt}+\beta_{3}\ln Dist_{ij}+\beta_{4}\ln POP_{jt}\\ +\beta_{5}{\mathrm{lnDG}}_{\mathrm{ijt}}+\beta_{6}\ln EC_{ijt}+\varepsilon_{ijt} \end{array}$$ (7)

Land area in ASEAN countries is added to the gravity model as a proxy variable for national resource endowments. In general, the higher a country’s resource endowment, the more self-sufficient it is internally and the less dependent it is externally. To ensure data continuity, the product of the Brent spot crude oil price and the size of the ASEAN countries are used as the resource endowment factor. The new gravity model is shown in Eq (8).


$$\begin{array}{l} lnT_{ijt}=\beta_{0}+\beta_{1}lnGDP_{t}+\beta_{2}lnGDP_{jt}+\beta_{3}\ln Dist_{ij}+\beta_{4}\ln POP_{jt}+\beta_{5}{\mathrm{lnDG}}_{\mathrm{ijt}}\\ +\beta_{6}\ln EC_{ijt}+\beta_{7}\ln Land_{j}+\varepsilon_{ijt} \end{array}$$ (8)

The signing of the "One Belt, One Road" cooperation agreement has greatly promoted the process of China-ASEAN multilateral trade liberalization. The signing of the "Belt and Road" cooperation agreement could greatly facilitate the process of China-ASEAN multilateral trade liberalization, which could lead to a significant reduction in bilateral trade tariffs and thus a significant increase in trade volumes. The construction of the China-ASEAN Free Trade Area will reduce trade barriers and enhance trade exchanges and development. The dummy variables BR and FT are added to the gravity model respectively to obtain Eq (9).


$$\begin{array}{l} lnT_{ijt}=\beta_{0}+\beta_{1}lnGDP_{t}+\beta_{2}lnGDP_{jt}+\beta_{3}lnDist_{ij}+\beta_{4}\ln POP_{jt}\\ \;+\beta_{5}\ln DG_{ijt}+\beta_{6}lnEC_{ijt}+\beta_{7}\ln Land_{j}+\beta_{8}BR_{ij}+\beta_{9}FT_{ij}+\varepsilon_{ijt} \end{array}$$ (9)

#### 3.4.3. Trade potential model

Trade potential model was used to estimate China’s trade potential with ASEAN countries. According to Shuai’s [32] and Batra’s [33] measure of trade potential, trade potential is the ratio of the actual value of trade to the theoretical estimate. The trade potential is calculated as in Eq (10):

$$TP_{ijt}=Trade_{ij}/Trade_{ij}{}^{*}$$ (10)

TP_ijt_ denotes the trade potential. Trade_ij_ denotes real trade volume between. Trade_ij_* denotes a theoretical estimate of trade potential as measured by the trade gravity model.

Liu Qingfeng and Jiang Shuzhu [34] set the trade potential level. When TP_ijt_≤0.8, bilateral trade is of the great potential type, indicating that there is great room for future enhancement for both sides in the future. When 0.8<TP_ijt_<1.2, bilateral trade is of the potential development type, this indicates that there is still room for growth in trade potential. When TP_ijt_≥1.2, bilateral trade is potentially reshaping, indicating that there is limited room for trade growth between the two sides and new trade growth points need to be explored.

## 4. Results and discussions

### 4.1. Data validation

Adding exogenous variables is a typical way of expanding the gravity model, but this study has been able to propose novel models due to the presence of multiple co-linearities and substantial uncorrelations among the independent variables. Given the significant uncorrelatedness between variables, this study conducted a correlation coefficient analysis on the variable data before introducing the specific model. The data were analyzed for correlation coefficients and treated accordingly before the introduction of the specific model. This study analyzes the correlation coefficients of the variables and treats them accordingly before introducing the specific model.

This study uses stata16 software to conduct a regression analysis on the panel data of trade between China and ten ASEAN countries for the period 2001–2021. Before conducting the regression analysis, the data are tested for smoothness, correlation, and cointegration, specifically: firstly, the natural logarithm method is used to mitigate the problem of heteroskedasticity; secondly, the correlation analysis test is conducted, and the results show that the correlation among all variables has a good correlation. The results are shown in Table 2. As can be seen from Table 2, the development of China-ASEAN trade is closely correlated with the total economic volume (GDP) of both sides, the distance from each other, the population size of ASEAN countries, land area, the signing of Belt and Road cooperation agreements and the establishment of a free trade area.

**Table 2:** Correlation of variables.

	lnT_ijt_	lnGDP_t_	lnGDP_jt_	lnDIST_ij_	lnPOP_jt_	lnDG_ijt_	lnEC_ijt_	lnLand_j_	BR	FT
**lnT**_**ijt**_	1									
**lnGDP**_**t**_	0.489***	1								
**lnGDP**_**jt**_	0.946***	0.362***	1							
**lnDIST**_**ij**_	0.355***	0.497***	0.356***	1						
**lnPOP**_**jt**_	0.575***	0.044	0.619***	-0.033	1					
**lnDG**_**ijt**_	0.034	0.409***	-0.043	0.344***	-0.584***	1				
**lnEC**_**ijt**_	0.106	0.128*	0.094	-0.111	0.349***	-0.105	1			
**lnLand**_**j**_	0.261***	0	0.448***	0.192***	0.658***	-0.373***	0.324***	1		
**BR**	0.340***	0.765***	0.219***	0.005	0.006	0.304***	0.11	-0.033	1	
**FT**	0.509***	0.835***	0.439***	0.426***	0.025	0.361***	-0.004	0.034	0.679***	1

The results of the collinearity test showed that the mean VIF values for all variables were 2.42, and the results are shown in Table 3. This indicates that the collinearity of the model is relatively low and can simply be carried out in the next step of the empirical analysis.

**Table 3:** Covariance test.

Variable	VIF	1/VIF
lnPOP_jt_	4.7	0.213
lnGDP_jt_	2.65	0.377
lnDG_ijt_	2.54	0.393
lnLand_j_	2.13	0.469
lnDIST_ij_	1.79	0.557
lnGDP_t_	1.78	0.562
lnEC_ij_	1.37	0.732
Mean	VIF	2.42

To avoid pseudo-regressions, a unit root test on the panel data is required to determine the stationarity of the variables. The LLC test and PP test were conducted for each variable separately. As can be seen from Table 4, the p-values of the LLC test and PP test for the variables lnT_ijt_, lnGDP_jt_, lnDIST_ij_, lnEC_ijt_, lnLand_j_ are all less than 0.05, i.e. the original hypothesis of the existence of a unit root is rejected at the 5% significance level, and the above data can be considered as having stationarity, however, the p-values of the LLC test and PP test for the variables lnGDP_t_, lnPOP_jt_, lnDG_ijt_ are all greater than 0.05 and a first-order difference j test is required to determine whether the data are stationary.

**Table 4:** Panel unit root test results.

Variables	lnT_ijt_	lnGDP_t_	lnGDP_jt_	lnDIST_ij_	lnPOP_jt_	lnDG_ijt_	lnEC_ijt_	lnLand_j_
**LLC test**	-1.288(0.0189)	20.130(0.4498)	44.262(0.0014)	-3.511(0.0002)	4.406(1.0000)	-0.616(0.2689)	33.667(0.0285)	-4.378(0.0000)
**PP test**	72.845 (0.0000)	24.781(0.2099)	35.551(0.0174)	32.030(0.0430)	18.516(0.5535)	19.598(0.4833)	-2.080(0.0188)	32.030(0.0430)
**stationary**	Yes	No	Yes	Yes	No	No	Yes	Yes

The variables lnGDP_t_, lnPOP_jt_, lnDG_ijt_ were subjected to first-order differences, and after passing the LLC test and PP test, it was found that the p-values were less than 0.05, and the data of the three variables were stable on the first-order differences, and the gravitational model could be carried out.

### 4.2. Econometric estimates of factors influencing the development of China-ASEAN bilateral trade

Panel data were analyzed using several types of regression models, including mixed regressions, fixed-effects regressions, and random-effects regressions, to estimate the factors influencing the development of China-ASEAN bilateral trade. The results from those regressions are displayed in Table 5.

**Table 5:** Regression results from three models.

	Ols	FE	RE
D1lnGDP_t_	1.608*	0.292	0.700
	(0.886)	(0.468)	(0.640)
lnGDP_jt_	1.100***	1.237***	1.095***
	(0.030)	(0.083)	(0.035)
lnDIST_ij_	-0.095	0.146	0.117
	(0.121)	(0.091)	(0.098)
D1lnPOP_jt_	8.504	8.182**	7.962*
	(5.821)	(3.334)	(4.487)
D1lnDG_ijt_	-0.018	0.007	-0.001
	(0.067)	(0.034)	(0.047)
lnEC_ijt_	-0.005	-0.019	-0.001
	(0.015)	(0.026)	(0.017)
lnLand	-0.012	0.000	-0.009
	(0.024)	(.)	(0.029)
BR	0.629***	0.396***	0.518***
	(0.136)	(0.082)	(0.098)
FT	0.096	0.098	0.136
	(0.148)	(0.081)	(0.109)
_cons	-10.676***	-16.887***	-13.060***
	(1.450)	(1.552)	(1.231)
N	200.000	200.000	200.000
r^2^	0.915	0.919	
r^2^_a	0.911	0.911	

As can be seen from Table 5, all variables passed the 1% significance level test for all three regression models. To determine the optimal regression model, the F-test, BP-test, and Hausman test were conducted to determine the optimal regression model. The results of the tests are shown in Table 6.

**Table 6:** F test, BP test, and Hausman test.

	Stata test results	Conclusion
F test	Prob>F = 0.0000	FE>Ols
BP test	Prob>Chibar2 = 0.0000	RE>Ols
Hausman test	Prob>Chibar2 = 0.9820	Ols>FE

Therefore, the final random effects model is chosen and the regression model is expressed as Eq (11):

$$\begin{array}{c} lnT_{ijt}=‐13.060+.700D1lnGDP_{t}+1.095lnGDP_{jt}+0.117\;lnDist_{ij}+7.962D1\ln POP_{jt}\\ ‐0.001D1\ln DG_{ijt}‐0.001\;lnEC_{ijt}‐0.009\;\ln Land_{j}+0.518BR_{ij}+0.136\;FT_{ij} \end{array}$$ (11)

Hence, holding other factors unchanged (ceteris paribus), the coefficient estimates (Eq 11) indicate that:

The GDP per capita of China and ASEAN countries both significantly contribute to the development of bilateral trade, with the coefficient of GDP per capita of China (0.700) being significantly lower than that of ASEAN countries (1.095), indicating that ASEAN economic development contributes a greater share of bilateral trade. The regression coefficient of the distance is positive and has a significant effect on the development of China-ASEAN trade, implying that physical distance between China and ASEAN countries does not limit the bilateral development of trade exchanges due to their geographical proximity and also proving that international logistics such as sea and air transport are well developed in East Asia [35].

An increase in the population size of ASEAN countries can contribute significantly to the development of China-ASEAN bilateral trade. The increase in the population size of ASEAN countries not only brings about an expansion of domestic demand but also raises the level of imports, which has a two-way effect on bilateral trade [36].

Although the regression coefficients of resource endowment per capita and the size of ASEAN countries are both negative, the results were in line with the study hypothesis. This indicates that the closer the living standards of the people in China and ASEAN countries are, the more similar the demand preferences are, and the trade between the two countries is very likely to increase.

The regression coefficient of the exchange rate between the RMB and the currencies of ASEAN countries is negative. The results were in line with the study hypothesis, indicating that the appreciation of the RMB will weaken the development of bilateral trade, but the impact is not very significant, indicating that China and ASEAN countries should maintain the stability of the exchange rate to maintain the healthy development of trade [37].

The two dummy variables: the signing of the Belt and Road Cooperation Agreement and the construction of the Free Trade Zone, both have a significant positive impact on trade. This indicates that the construction of the Belt and Road and the Free Trade Zone is conducive to the reduction of tariffs and quantitative restrictions in commodity trade, allowing the free flow of goods between member countries, reducing trade barriers, and promoting trade between the two sides.

The trade volume of ASEAN countries is divided into four groups in descending order, and their heterogeneity is tested. The following results can be obtained after group regression: (i) the higher the trade volume of ASEAN countries, the better the significance of their economic aggregates; (ii) the higher the economic volume, the lower the suppression of the country’s trade. The regression results are consistent with the expected hypothesis.

### 4.3. Estimates of the China-ASEAN trade potential

The China-ASEAN trade potential index is estimated by comparing the actual value of bilateral trade volume to the theoretical value of the model, as illustrated in Table 7 for the ten Chinese and ASEAN countries from 2012 to 2021.

**Table 7:** China-ASEAN trade potential index 2012–2021.

	2012	2013	2014	2015	2016	2017	2018	2019	2020	2021	Average value	Type of potential
Malaysia	1.036	1.042	1.009	1.019	1.015	1.013	1.010	1.020	1.030	1.032	1.023	Expansion
Philippines	0.995	0.994	1.001	0.978	0.980	0.979	0.978	0.982	0.987	0.989	0.986	Expansion
Thailand	1.011	1.009	0.986	0.994	0.995	0.988	0.984	0.987	0.998	1.004	0.996	Expansion
Singapore	1.015	1.021	0.997	1.004	0.997	1.000	0.993	0.998	1.012	1.014	1.005	Expansion
Cambodia	1.100	1.062	1.057	1.069	1.066	1.065	1.071	1.084	1.092	1.104	1.077	Expansion
Laos	1.147	1.111	1.128	1.108	1.084	1.090	1.095	1.106	1.106	1.124	1.110	Expansion
Myanmar	1.013	1.030	1.051	1.029	1.022	1.023	1.023	1.039	1.031	1.041	1.030	Expansion
Vietnam	1.298	1.303	1.273	1.292	1.275	1.275	1.284	1.284	1.299	1.311	1.289	Remodeling
Brunei	0.975	0.986	0.963	0.973	0.936	0.947	0.979	0.949	0.999	1.004	0.971	Expansion
Indonesia	1.035	1.049	1.064	1.044	1.032	1.031	1.048	1.044	1.053	1.068	1.047	Expansion

As shown in Table 7, the trade potential between China and Vietnam falls into the category of potential re-modelling, indicating that both sides are currently utilizing their trade potential to the greatest extent possible, that trade growth space is limited, and that new trade opportunities must be discovered.

The trade potential index between China and nine ASEAN countries, excluding Vietnam, is in the potential-exploiting category, indicating that the potential has not been fully utilized by both sides and that there is still room for growth in the scale of trade between the two countries.

### 4.4. Estimates of the China-ASEAN trade structure

The China-ASEAN trade structure is exceptionally rich after more than 20 years of bilateral trade and economic development, with bilateral exports and imports covering all commodities in the ten major categories of the United Nations Standard International Trade Classification of Commodities (SITC Rev. 3). Table 8 illustrates the status of China’s and ASEAN’s merchandise categories and proportions in 2021. China and ASEAN are also more concentrated in terms of their respective import and export categories. The share of Chinese machinery exports and transport equipment to ASEAN was 42.24%, with the largest share of exports of "metal products" and "machinery and equipment products", which together accounted for nearly 70% of total exports, of which metal products accounted for another 60% of both products. The share of metal products in both products reached 60%. Among the products other than "metal products" and "machinery and equipment products", "electrical and electronic products", "means of transport", "instruments and apparatus", and "machinery and equipment products" had the largest export shares. Other than "metal products" and "machinery and equipment products," the proportion of exports of "electrical and electronic products," "means of transport," and "instruments and apparatus" ranged from 5% to 15%. China’s exports of electromechanical products to ASEAN accounted for 44.53%, mainly concentrated in automobiles, automobile chassis, auto parts and components, and other products, highlighting China’s competitive advantage in the automotive industry, especially in the field of new energy vehicles. In terms of the categories of goods, the focus of bilateral trade in both imports and exports is SITC7 (machinery and transport equipment), but the products traded between China and ASEAN do not clash but rather complement each other in their advantageous industries [38].

**Table 8:** China’s import and export commodity categories and share to ASEAN countries in 2021.

	Export		Import	
	SITC	Account for	SITC	Account for
2021	SITC7	42.24%	SITC7	44.53%
	SITC6	23.23%	SITC3	14.20%
	SITC8	15.69%	SITC6	9.56%
	SITC5	9.46%	SITC2	7.94%
	SITC0	3.97%	SITC5	7.81%
	SITC3	3.01%	SITC8	5.62%
	SITC9	1.48%	SITC0	5.19%
	SITC2	0.71%	SITC9	2.62%
	SITC1	0.13%	SITC4	2.44%
	SITC4	0.09%	SITC1	0.10%

**Source:** UN Comtrade Database (SITC, Rev3).

In terms of the types of goods traded between the two sides, China and ASEAN have a richer trade structure, both sides are expanding in their advantageous industries, there is no trade competitiveness, and the trade products are highly complementary [39, 40]. Table 9 shows the main commodity categories and shares in 2021. Thailand, Vietnam, Indonesia, Malaysia, Singapore, and the Philippines are the countries with the highest share of trade in imported Chinese SITC7 (machinery and transport equipment), while Vietnam also has the highest share of trade in imported Chinese SITC6 (manufactured goods by raw materials) and SITC8 (miscellaneous products). From an ASEAN export perspective, Thailand, Vietnam, Singapore, Malaysia, Thailand, and the Philippines are the countries with the highest share of trade in exports of SITC7 (machinery and transport equipment), while Malaysia and Indonesia also have a relatively high share of exports of SITC6 (manufactured goods by raw materials) to China.

**Table 9:** China’s import and export commodity categories and share to major ASEAN member countries in 2021.

		Export			Imported	
	Country	Commodity Classification	Proportion		Commodity Classification	Proportion
2021	Vietnam	SITC7	13.36%	Vietnam	SITC7	15.34%
	Vietnam	SITC6	7.16%	Malaysia	SITC7	12.60%
	Malaysia	SITC7	7.16%	Thailand	SITC7	7.28%
	Singapore	SITC7	6.45%	Malaysia	SITC3	6.34%
	Thailand	SITC7	5.67%	Indonesia	SITC3	5.75%
	Indonesia	SITC7	4.90%	Singapore	SITC7	4.38%
	Vietnam	SITC8	3.69%	Philippines	SITC7	4.24%
	Philippines	SITC7	3.61%	Indonesia	SITC6	4.12%
	Malaysia	SITC8	3.56%	Thailand	SITC0	2.55%
	Philippines	SITC6	3.43%	Singapore	SITC5	2.48%

Data source: UN Comtrade Database (SITC, Rev3).

## 5. Conclusions and recommendations

### 5.1. Conclusions

This study is based on data relating to 10 partner countries in China and ASEAN for the period 2001–2021. Based on the gravity model empirically analyzing the influencing factors of China-ASEAN bilateral trade, the study found that the GDP of China and ASEAN countries, the distance between trading countries, and the population size of ASEAN countries have a significant positive influence on bilateral trade. However, per capita resource endowment, the exchange rate between RMB and ASEAN countries’ currencies, and the land area of each ASEAN country have a negative impact on bilateral trade, but the regression coefficients are small and the negative impact is not significant. In terms of overall trade potential, China has great potential for trade development with 9 out of 10 ASEAN countries. The average value of the China-ASEAN Trade Potential Index is 1.05, which means that China-ASEAN trade has not yet reached saturation and there is room for further expansion.

With the shift of the world’s economic center of gravity in the direction of Asia following COVID-19, China and ASEAN countries should seize the opportunity to strengthen their comprehensive strength and economic aggregates and further develop China’s constructive role in the regional organization. The gravity model shows that the coefficients of economic size (0.700) and (1.095) on both sides of the trade have a greater impact on China-ASEAN trade flows, indicating that economic size will have a positive impact on bilateral trade. The impact of population size in ASEAN countries (7.962) on bilateral trade is significant and positive. The signing of the Belt and Road Initiative and the construction of a free trade zone has had a positive effect on the development of bilateral trade.

I must acknowledge some limitations of my study. With the development of institutional economics, increasing attention has been paid to the impact of formal and informal systems on international trade flows. In my future research, I will add legal system, contract enforcement guarantee system, security of property rights, and other factors that affect the expectations of both parties to a transaction about the security of the transaction to the trade gravity model.

In addition, in my subsequent research, I will focus on a series of explanatory variables reflecting the quality of a country’s institutions, such as the Trade Policy Index, the Government Fiscal Burden Index, the Government Intervention Index, the Monetary Policy Index, the Capital Flows and Foreign Investments Index, the Banking and Finance Index, the Remuneration and Prices Index, the Property Rights Index, the Regulatory Index, and the Black Market Activity Index, and I will explore and analyze the policy impacts of the institutional factors on the development of China-ASEAN trade.

### 5.2. Recommendations

Based on the above findings, the following recommendations are made:

Firstly, promote the construction of infrastructure in trade information and logistics on both sides. Accelerate the projects related to the ASEAN Connectivity Master Plan 2025 between China and ASEAN in terms of facility connectivity construction, open up bottlenecks in infrastructure development in ASEAN countries in terms of railways, highways, ports, and information, and promote the convergence of development plans in southwest China with those of neighboring countries, enhance the level of trade connectivity between China and ASEAN, and create a favorable environment for promoting the development of border trade, logistics networks, etc. The two sides should strengthen communication and consultation on trade policies and actively promote the development of border trade and logistics networks. The two sides will strengthen communication and consultation on trade policies, actively promote the implementation of the relevant contents of the “Belt and Road” agreement, and strengthen consultation with ASEAN on trade regimes in technology and services, further remove non-tariff barriers that exist between China and ASEAN, weaken the hindering effect of trade barriers on trade, and create a favorable institutional environment for bilateral trade.

Secondly, accelerate the work related to the implementation of the Belt and Road and FTA-related agreements, and open up a win-win space for China-ASEAN contracting parties in international trade cooperation. Through the implementation of the Belt and Road and the Free Trade Area, tariffs and trade barriers between the contracting parties will be further broken down, the division of labor in the industrial chain will be extended while the costs for domestic enterprises of the contracting parties to participate in international trade will be further reduced, and the trade between the China-ASEAN contracting parties will be complementary and mutually beneficial.

Thirdly, promote and strengthen the distribution of labor between China and ASEAN countries through high-level visits to better complement each other’s strengths and reduce over-dependence on countries and markets such as Russia, the US, the UK, and Germany. The Trade Potential Index shows that China’s trade potential with nine ASEAN countries, excluding Vietnam, is huge and there is plenty of room for trade development, while China-Vietnam needs to develop new areas of trade expansion. Based on maintaining the original export volume, China and ASEAN countries took advantage of their resource endowments to actively explore new trade growth points and increase exports of more complementary products, achieving a steady increase in trade volume.

This study provided a clear insight into the factors that influence bilateral trade flow. GDPs of countries, Geographical distance between countries, Population size, Resource endowments per capita, Exchange rate between RMB and ASEAN, Land area in ASEAN countries, "One Belt, One Road" cooperation agreement, China-ASEAN Free Trade Area as key factors in bilateral trade flow. As a consequence, a conceptual framework for China-ASEAN trade status and trade potential, which emphasizes that FTAs and Belt and Road agreements will be leverage points for future interventions and will be an important direction for future research.

## Supporting information

S1 Data (XLSX)
